# Anti-glycan IgM repertoires in newborn human cord blood

**DOI:** 10.1371/journal.pone.0218575

**Published:** 2019-07-31

**Authors:** Li Xia, Jeffrey C. Gildersleeve

**Affiliations:** Chemical Biology Laboratory, Center for Cancer Research, National Cancer Institute, National Institutes of Health, Frederick, Maryland, United States of America; Duke University School of Medicine, UNITED STATES

## Abstract

Natural antibodies are an innate-like subset of serum antibodies involved in host defense, tumor surveillance, homeostasis, and autoimmunity. Defining the natural antibody repertoire is critical for identifying biomarkers, developing vaccines, controlling and preventing autoimmunity, and understanding the development and organization of the immune system. While natural antibodies to protein antigens have been studied in depth, little is known about natural antibodies to carbohydrate antigens. To address this, we profiled IgM from umbilical cord blood and matched maternal sera on a glycan microarray. Since standard methods to detect maternal contamination in cord serum did not have sufficient sensitivity for our study, we developed a highly sensitive microarray-based assay. Using this method, we found that over 50% of the cord samples had unacceptable levels of maternal contamination. For the cord samples with high purity, anti-glycan IgM antibodies were prevalent and recognized a broad range of non-human and human glycans. Using principal component analysis and hierarchical clustering, cord IgM repertoires showed a high degree of similarity with each other but were distinct from maternal IgM repertoires. Our results demonstrate that many anti-glycan antibodies in human serum are natural antibodies and provide new insights into the development of anti-glycan antibody repertoires.

## Abbreviations

The abbreviations used are: BG-A, blood group A; BG-B, blood group B; HSA, human serum albumin. LeX, Lewis X; LeC, Lewis C; SSEA4, stage-specific embryonic antigen 4; SSEA5, stage specific embryonic antigen 5. Monosaccharide residues are abbreviated as follows: Fuc, fucose; Gal, galactose; GalNAc, N-acetyl-galactosamine; Glc, glucose; GlcNAc, N-acetyl-glucosamine; Neu5Ac, N-acetylneuraminic acid. Abbreviations for all microarray components can be found in Table 1 and the Supporting Information S1 File.

**Table 1:** Summary of cord IgM signals for selected array components.

Abbreviation^1^	Description	Mean^2^ signal	Max^2^ signal	# of samples with IgM ≥8.8 (3-fold > bkg)
BG-H2	Fucα1-2Galβ1-4GlcNAcβ	10.1	11.3	5
BG-H4	Fucα1-2Galβ1-3GalNAcβ	10.3	13.4	6
BG-H5	Fucα1-2Galβ1-3Galβ	10.4	13.5	6
Gb5/SSEA3	Galβ1-3GalNAcβ1-3Galα1-4Galβ	10.1	11.8	5
Lac	Galβ1-4Glc	10.0	10.9	8
LacNAc (trimeric)	Galβ1-4GlcNAcβ1-3Galβ1-4GlcNAcβ1-3Galβ1-4GlcNAc	11.0	12.7	8
LDN	GalNAcβ1-4GlcNAc	10.5	13.0	7
GNLacNAc	GlcNAcβ1-3Galβ1-4GlcNAc	12.0	14.2	8
LNT-2	GlcNAcβ1-3Galβ1-4Glc	12.1	13.1	8
P1	Galα1-4Galβ1-4GlcNAc	11.6	14.2	8
3'-sulpho-LeX	3-SO_3_-Galβ1–4[Fucα1–3)GlcNAc-	10.3	12.1	7
3'Neu5Ac(9Ac)-LeC	Neu5Ac(9Ac)α2-3Galβ1-3GlcNAc	10.0	11.2	7
6'Neu5Ac-LacNAc (dimeric)	Neu5Acα2-6Galβ1-4GlcNAcβ1-3Galβ1-4GlcNAc	12.1	13.7	8
**GD2**	Neu5Acα2-8Neu5Acα2–3[GalNAcβ1–4]Galβ1-4Glc	9.9	13.1	5
GT2	Neu5Acα2-8Neu5Acα2-8Neu5Acα2–3[GalNAcβ1–4]Galβ1-4Glc	9.7	12.4	5
CT/Sda	Neu5Acα2–3[GalNAcβ1–4]Galβ1-4GlcNAc	10.5	12.0	8
**Muc1-Tn15**	GVTSAPDTRPAPGS-(GalNAcα)T-APPA	14.9	16.1	8
**Muc1-Tn8**	GVTSAPD-(GalNAcα)T-RPAPGSTAPPA	15.3	16.2	5
**Muc1**	GVTSAPDTRPAPGSTAPPA	13.5	14.6	5
**GTSSA-TF(Ser)-TF(Thr)-GHATPLPVTD**	GTSSA-(Galβ1-3GalNAcα)S-(Galβ1-3GalNAcα)T-GHATPLPVTD	10.6	13.0	8
**Ac-APGS-Tn(Thr)-APPA-G**	Ac-APGS-Tn(Thr)-APPA-G-BSA	11.5	12.9	7
**Ac-Tn(Ser)-Tn(Ser)-Tn(Ser)-G**	Ac-(GalNAcα)S-(GalNAcα)S-(GalNAcα)S-G	10.7	14.3	6
**Ac-TnThr)-G**	Ac-(GalNAcα)T-G	11.0	12.0	8
**SAPD-Tn(Thr)-RPAP**	SAPD-(GalNAcα)T-RPAP	15.5	16.8	8
**SAPDTRPAP**	SAPDTRPAP	13.4	15.5	8
Cellotriose	Glcβ1-4Glcβ1-4Glcβ; non-human	9.9	12.0	6
Chitotriose	GlcNAcβ1-4GlcNAcβ1-4GlcNAcβ; non-human	12.0	13.9	8
Rha-a	Rha-α; non-human	10.3	13.7	7
DNP-BSA	dinitrophenyl-bovine serum albumin	17.3	17.7	8
**Alpha-fetoprotein**	Alpha-fetoprotein	11.1	14.0	7
BSM	Bovine submaxillary mucin	10.8	12.2	8
fetuin (human)	fetuin (human)	12.1	12.9	6

^**1**^Components listed in the following order: neutral human glycans, anionic human glycans, glycopeptides/peptides, non-human glycans, and glycoproteins. Components in bold are known tumor-associated antigens.

^**2**^All signals are on a log_2_ scale. For full list of cord signals, see Table A in S1 File and Supporting Excel File, S2 File. For additional information about each component, see the Supporting Excel File, S2 File.

## Introduction

Serum antibodies play key roles in host defense and homeostasis, but mis-regulation can lead to complications such as autoimmunity. Some antibodies are produced upon exposure to pathogens, microbes, or vaccine antigens as part of our adaptive immune responses. Others, referred to as “natural antibodies,” are an innate-like subset of antibodies produced by mammals in the absence of external antigen stimulation.[1–9] Natural antibodies are maintained at consistent levels throughout life, and they can affect one’s health in a variety of ways. Some react with foreign antigens and are important for host defense against bacteria, viruses, and other microorganisms. Others bind self-antigens and contribute to removal of apoptotic cells, tumor cells, senescent cells, and associated cellular debris. Distinguishing antibodies that are part of our adaptive response versus those that are encoded as part of our natural antibody repertoire is critical when using serum antibodies as diagnostics/biomarkers, stimulating and monitoring antibody responses to vaccines, controlling/preventing autoimmune reactions, and understanding the development of our immune system. At present, the natural antibody repertoire in humans is not fully characterized.

Human serum contains a rich and diverse collection of carbohydrate-binding antibodies, and many of these antibodies exhibit characteristics commonly associated with natural antibodies. For example, many anti-glycan IgG and IgM antibodies in serum are expressed at consistent levels for years.[10, 11] In addition, many anti-glycan antibodies are thought to have modest affinity and be polyspecific, traits that are associated with natural antibodies. Therefore, it has been hypothesized that many of the anti-glycan antibodies found in serum are natural antibodies. Unfortunately, it has been difficult to determine which, if any, anti-glycan antibodies are natural antibodies.

One defining feature of natural antibodies is production in the absence of apparent external antigen stimulation. Natural antibodies can be present at birth, so umbilical cord blood has been a key resource for gaining insights into natural antibody repertoires and reactivities (for some examples, see [12–18]). While IgG antibodies from the mother are transferred to the baby, IgM antibodies are not. Therefore, IgM populations in cord blood are derived from the baby and are considered natural antibodies. The average serum concentration of IgM in newborns is about 100 μg/mL.[19] While the repertoire of natural IgM antibodies in cord blood to protein antigens has been studied in depth,[12–16] much less is known about natural antibodies to carbohydrate antigens. In fact, the presence of anti-glycan IgM antibodies at birth is still a matter of debate. For example, IgM to a β-glucan and chitosan have been observed in cord blood,[15] but others reported that anti-glycan antibodies are not present during the first few weeks of life.[5]

To further test the hypothesis that many of the anti-glycan antibodies found in serum are natural antibodies, we used glycan antigen microarrays to profile IgM antibody repertoires in cord blood and maternal blood. During these studies, it became apparent that even very low levels of maternal contamination (0.1%) could have a major impact on the IgM signals observed in cord blood for anti-glycan antibodies. We developed a highly sensitive method for detecting maternal contamination and used it to identify cord blood samples with greater than 99.97% purity. High purity human cord samples were found to possess numerous anti-glycan IgM antibodies, a result that supports the hypothesis that many are natural antibodies.

## Materials and methods

### Serum samples

Publicly available, de-identified serum samples from 20 cord-maternal pairs were purchased from ProMedDx (Norton, MA). Sample collection and handling followed standard protocols.[20] Samples were tested in accordance with FDA regulations and found to be negative for HIV ½ AB, HCV AB, and non-reactive for HBSAG, HIV-1 RNA, HCV RNA, and STS. All sera were aliquoted and stored at −80 °C until use.

### ELISA for measuring serum IgM and IgA levels

IgM and IgA levels in cord and maternal samples were measured with human IgM and IgA Ready-set-go kits (Affymetrix eBioscience), respectively. Cord samples were profiled at 1:500 dilution, and maternal samples were profiled at 1:10,000 dilution. Each sample was measured in duplicate. One maternal serum sample had IgA levels of 1.5 μg/mL. Since this level is >1000 lower than expected and more consistent with a cord sample, we concluded that it was likely mis-labeled. This sample and its cord pair were excluded from further analysis.

### Microarray fabrication and assay

The glycan microarrays were fabricated as previously reported.[21, 22] Glycans and glycopeptides were conjugated to either bovine serum albumin (BSA) or human serum albumin (HSA) to produce neoglycoproteins, which were then printed on the array surface. Some glycans were conjugated at high (10–20 per molecule of albumin) and low (3–7 per molecule of albumin) valencies, as denoted by the number following the array component name, to produce different densities on the array surface. The microarray contained 503 array components on Array A503 and 411 components on Array A411. Both versions of the array included a variety of human glycans (*N*-linked glycans, and *O*-linked glycans, and glycan portions of glycolipids), non-human glycans, glycopeptides, and glycoproteins. Each array component was printed in duplicate to produce a full array, and 16 copies of the full array were printed on each slide. Prior to the assay, slides were fitted with a 16-well module/gasket (Sigma-Aldrich) to allow 16 independent assays on each slide. In the assay,[23] arrays were blocked with 3% BSA in PBS buffer (200 μL/well) overnight at 4 °C, then washed six times with PBST buffer (PBS with 0.05% v/v Tween 20). Cord and maternal serum samples diluted at 1:5 in 3% BSA and 1% HSA in PBST were added onto each slide (60 μL/well). To minimize technical variations, all samples were assayed in duplicate, and each paired cord and maternal sera were run on the same slide. After agitation at 100 rpm for 4h at 37 °C, slides were washed six times with PBST (200 μL/well). The bound serum antibodies were detected by incubating with DyLight 549 anti-Human IgA and/or DyLight 649 anti-human IgM (Jackson ImmunoResearch) at 2 μg/L in PBS buffer with 1% BSA and 3% HSA (100 μL/well) at 37 °C for 2h. After washing with PBST seven times (200 μL/well), the slides were dried by centrifugation at 1000× rpm (200 *g*) and then scanned with a Genepix 4000B microarray scanner (Molecular Devices) or InnoScan 1100 at 10 μm resolution or finer. The fluorescence intensity of each array spot was quantified with GenePix 7.0 software (Molecular Devices), and the average of 4 spots for each component was determined (averaged across 2 replicate spots from 2 separate array wells). Analysis of reproducibility[24] and validation of the array with numerous antibodies and lectins have been previously published.[25–28] Representative images illustrating the quality of the data can be found in the Supporting Information (see Figure A in S1 File). Full microarray data can be found in the Supporting Excel file, S2 File. The 8 pure cord samples were also evaluated on a different version of our microarray containing 411 components. Data from the 503-component array and the 411-component array were highly correlated (see Supporting Information, Figure B in S1 File) indicating good reproducibility of the data.

### Assay for detecting maternal contamination

For addition of maternal sera to affect the overall antibody profile, the final assay concentration of maternal serum must be sufficient to produce signals and the maternal and cord anti-glycan antibody repertoires must have differences that can be detected. We assayed cord samples at a dilution of 1:5; therefore, adding 0.01% maternal serum corresponds to a final assay dilution of 1:50,000. Likewise, adding 0.1% maternal serum corresponds to a final dilution of 1:5,000. From our experience, normal sera produce very few signals (if any) in our assay at 1:50,000, whereas we might expect 20–60 signals at 1:5000. Therefore, our initial test with pair 3 and pair 11 included 0.01, 0.1 and 1% v/v of their corresponding matched maternal sera. Based on the initial results, all other samples were tested with 0.1% v/v of the corresponding maternal samples. Cord sera without maternal spiking were used for comparison. The cord-maternal mixtures (60 μL/well) were profiled on the glycan microarray and analyzed as described above.

### Statistical analysis

Pearson coefficients between cord and maternal samples were calculated in Excel. p-values were calculated using t test for correlation in Excel. Heatmaps, clustering analysis, principle component analysis and quantile normalization were carried out in Partek Genomics Suite 6.6. Hierarchical clustering was carried out for both samples and array components using average linkage and Euclidean distance metric.

## Results

### Evaluation of IgA levels in maternal/cord pairs

**Fig 1:**
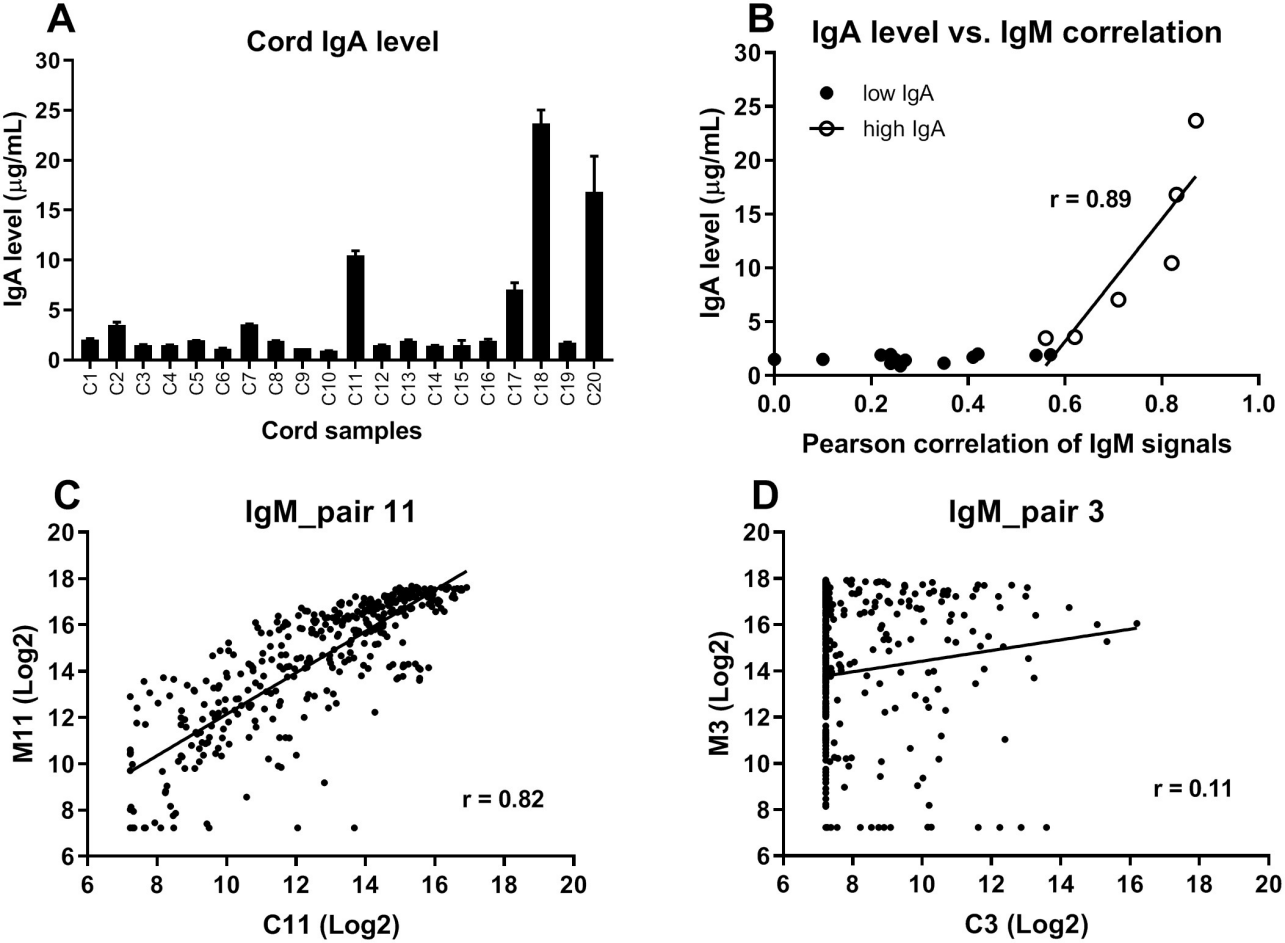
Relationship between cord IgA level and Pearson coefficients for cord-maternal IgM profiles. A) IgA levels in cord samples. B) Plot of IgA level (y-axis) versus Pearson correlation of cord and maternal IgM signals measured on the array (x-axis). Solid dots represent samples with low IgA level and circles represent samples with high IgA level. Linear regression was fitted using samples with high IgA level. Also shown are representative scatter plots of IgM signals for cord serum (x-axis) versus matched maternal serum (y-axis) for a contaminated cord sample (C) and pure cord sample (D).

Serum from cord samples can be contaminated with maternal blood.[29] The standard method to detect this contamination involves measuring IgA levels in cord serum.[30] Newborns produce very low levels of IgA, whereas the mean concentration of IgA in maternal blood is about 2–3 mg/mL.[31] To detect potential contamination, we measured IgA concentrations in all individual cord blood samples (Fig 1A). The average IgA level for the 20 cord samples was 4.4 μg/mL. The optimal cutoff for cord IgA levels remains under debate. Depending on the study, the cutoff has varied from 10 μg/mL[32] to 100 μg/mL,[33] with most using a cutoff around 30 μg/mL.[30] Among our samples, three (C11, C18, C20) had IgA concentrations greater than 10 μg/mL (10.5 μg/mL for C11, 23.7 μg/mL for C18, 16.8 μg/mL for C20). We concluded that these three samples were likely contaminated with maternal serum; however, it was not yet clear if this low level of contamination would be problematic for evaluating anti-glycan IgM antibodies.

### Glycan microarray profiling of serum IgM

Next, we profiled serum IgM from all 20 matched pairs of cord blood and maternal blood on our glycan microarray. The array contained 503 components, including a wide variety of *N*-linked glycans, *O*-linked glycans, glycolipid glycans, glycopeptides, and non-human glycans. In addition, the array also included some natural glycoproteins. Thus, the array would be capable of detecting a diverse assortment of anti-glycan antibody populations in serum.

For the three cord samples with IgA levels greater than 10 μg/mL (C11, C18, C20), the anti-glycan IgM profiles were highly correlated with their corresponding maternal samples (Pearson correlation coefficient *r* = 0.82, 0.83, 0.87, p<0.0001 for all). We have profiled hundreds of human serum samples from different subjects as well as samples from the same subjects over time, and the degree of correlation observed for these three cord/maternal pairs was far higher than expected for separate individuals.[10, 11, 24, 34] The correlation was also much higher than most other pairs (*r* = 0.002–0.71, median = 0.30; see also Figure C in S1 File). For these reasons, we concluded that most IgM signals in these cord samples were likely derived from maternal contamination and that we would need an IgA cutoff of at least 10 μg/mL.

Several other cord-maternal pairs had unusually high anti-glycan antibody correlations, even though the cord blood IgA concentrations were less than 10 μg/mL. Moreover, higher IgA levels corresponded to a better correlation of IgM signals between cord and maternal samples (Fig 1B). These results suggested that maternal IgM likely dominate the antibody profiles for these cord blood samples. Therefore, we hypothesized that anti-glycan antibody profiles are especially sensitive to maternal contamination and that even a very conservative IgA cutoff, such as 10 μg/mL, is not sufficient when studying anti-glycan antibodies in cord blood.

To evaluate the effects of maternal contamination more directly, we opted to add or “spike in” small amounts of maternal serum into matched cord blood samples. After adding maternal serum in samples, we could then measure changes in IgM signals and isolate the maternally derived signals. We selected two samples for the initial evaluation: one sample with IgA above 10 μg/mL and a high correlation with maternal profile (C11; IgA = 10.5 μg/mL Pearson correlation coefficient with maternal sample M11 = 0.82, see Fig 1C) and one sample with low IgA and a low correlation with its maternal sample (C3; IgA = 1.5 μg/mL Pearson correlation coefficient with M3 = 0.11, see Fig 1D).

**Fig 2:**
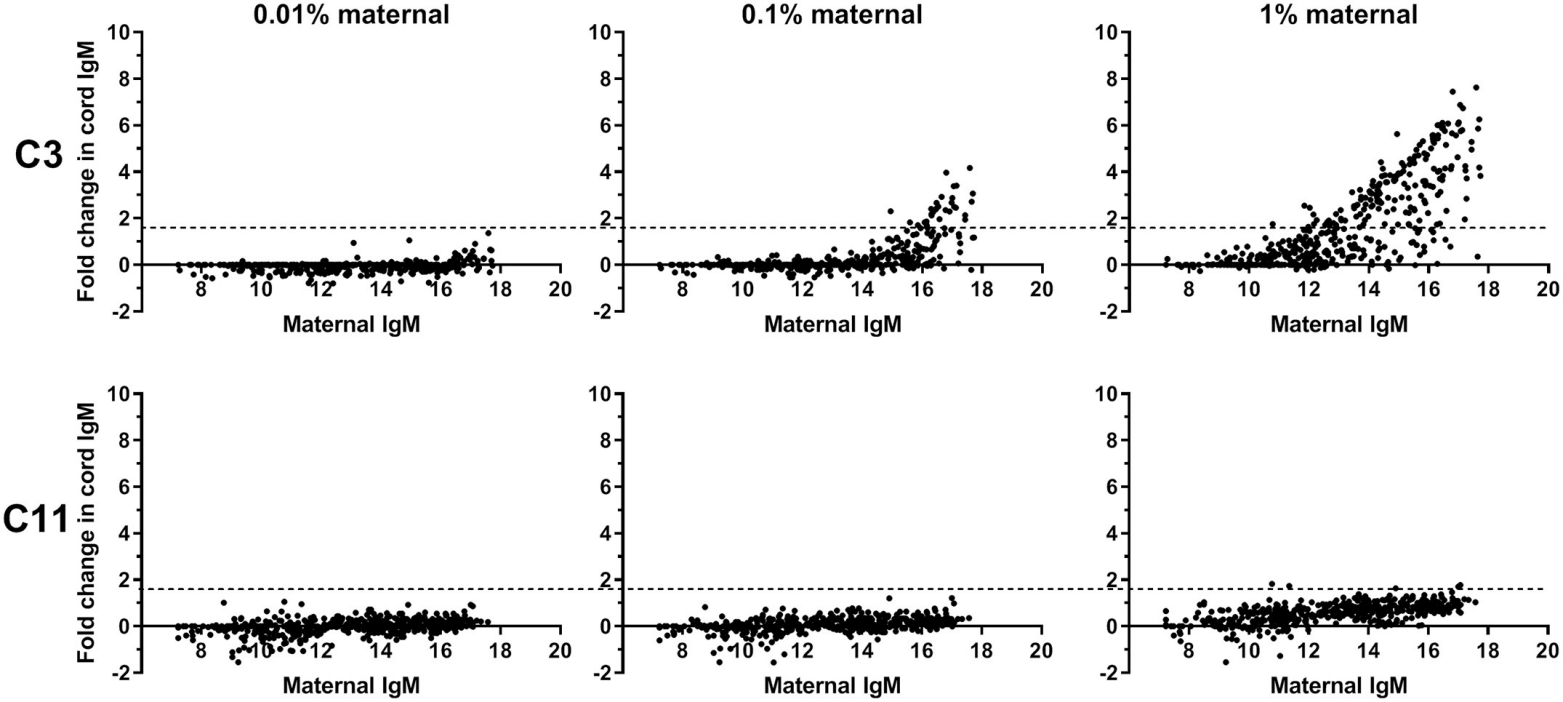
Evaluation of maternal contamination. Two pairs of cord-maternal sera were used. Pair #3 had dissimilar cord and maternal IgM profiles (top panel). Pair #11 had highly similar cord and maternal IgM profiles (bottom panel). Cord samples were spiked in with 0.01%, 0.1% and 1% of their corresponding maternal sera; IgM antibodies in the resulting mixture was assayed with our glycan microarray. X-axis: IgM signals in maternal sera (in Log2 scale); Y-axis: The change of IgM antibody signals in maternal-spiked cord relative to cord alone (in Log2 scale). Dotted line indicates a 3-fold cutoff.

We tested addition of 0.01%, 0.1%, and 1% maternal serum into each of the two cord samples and then evaluate effects using our microarray (Fig 2). When sample C3 was tested, IgM antibody signals significantly increased even after adding as little as 0.1% maternal serum (paired t-test, P<0.0001; 60 array components with a 2-fold or greater increase and 44 completely new signals). The magnitudes of the increases for various glycans directly correlated with the signal intensity found in the matched maternal sample. Addition of 0.01% matched maternal samples did not significantly change the cord IgM profile. Based on these results, we concluded that the purity of this cord sample was greater than 99.9%, as adding 0.1% matched maternal serum was enough to produce significant changes in the antibody profiles. In comparison, when we tested sample C11, the IgM antibody repertoire/profile remained essentially unchanged even after adding 1% matched maternal serum (i.e. slight increase in signal intensity but no new signals). The lack of change in the antibody profile after adding 1% matched maternal serum suggests that this cord sample was contaminated with at least 1% maternal serum. For comparison, an IgA level of 10 μg/mL has previously been estimated to correspond to 0.8% maternal serum in a cord sample,[30] so our estimate derived from the array results is consistent with estimates based on IgA levels.

**Fig 3:**
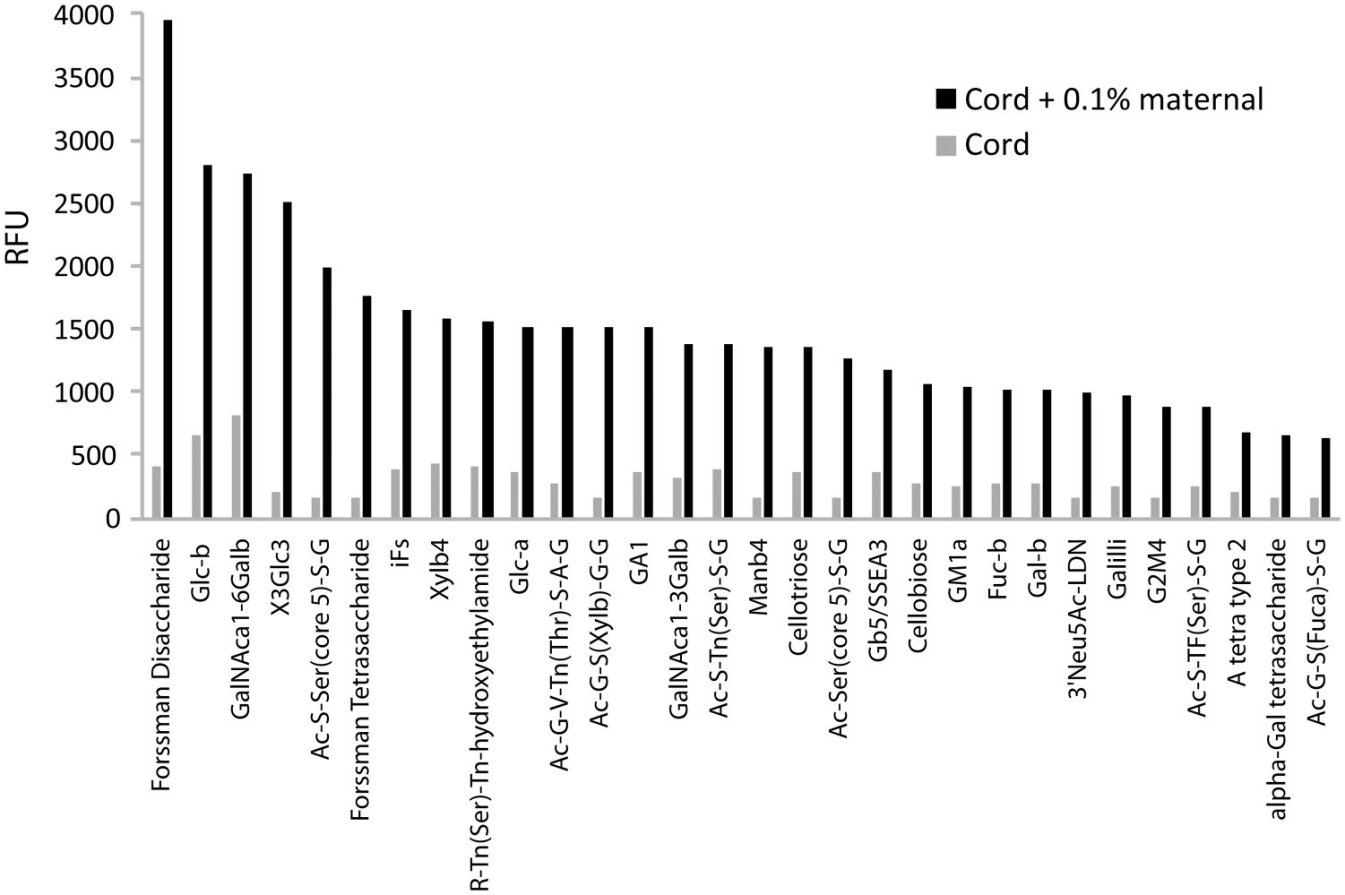
Impact of 0.1% maternal contamination on cord IgM antibody profiles. The bar graph compares RFU signals for 30 array components before and after addition of 0.1% maternal serum sample M4 into cord sample C4. All signals below 150 are set to 150 (floor = 150 RFU).

These findings indicate that even very low levels of maternal contamination can significantly affect anti-glycan IgM in cord blood. As illustrated in the example shown in Fig 3, even 0.1% maternal contamination can give rise to numerous anti-glycan IgM signals on the array that are either not actually present or had weak signals in the cord blood sample. For example, the signal for anti-Forssman tetrasaccharide IgM from the cord sample was below our detection limit (all RFU values below 150 are set to 150, our floor value) but it increased to 1700 after adding 0.1% maternal serum.

To reliably detect anti-glycan antibodies derived specifically from the infant, we needed cord samples with very low maternal contamination (i.e. <0.1%). Unfortunately, the standard method of measuring IgA levels has a lower limit of detection around 1% maternal contamination. We anticipated that the maternal spike-in experiment described above could serve as a more sensitive alternative to IgA measurements to detect contamination of cord blood. We opted to compare cord IgM profiles before and after addition of 0.1% matched maternal serum for the remaining cord/maternal pairs. As an estimate, if a cord sample had 0.05% maternal contamination, adding 0.1% more maternal serum should produce at most a 3-fold increase in any given array signal. So, increases above 3-fold would signify that the original cord sample had less than ~0.05% contamination. Similarly, increases above 4-fold would indicate that the original cord sample had less than ~0.033% contamination. As a caveat for this approach, if the matched maternal sample and cord sample have very similar or identical antibody profiles, this approach would not work (i.e. the cord sample would appear to be contaminated with maternal serum). In our experience so far, however, this situation appears to be uncommon.

We note that a contamination level of 0.033% is near the lower limit of sensitivity for our method. We profiled the cord samples at a dilution of 1:5. Therefore, 0.033% maternal contamination would correspond to a dilution of 1:15,000 for the maternal components. Based on extensive serum profiling of normal adult samples, a dilution of 1:15,000 produces relatively few signals on the array, and those signals are weak. For example, we would expect a typical adult sample to have about 10 array components with signals in the range of 200–500 RFU at a dilution of 15,000. We would expect another 30–40 signals in the 100–200 RFU range. We would not expect any signals over 1000. Thus, maternal contamination of 0.033% or less would have a minimal or no effect on cord profiles.

**Fig 4:**
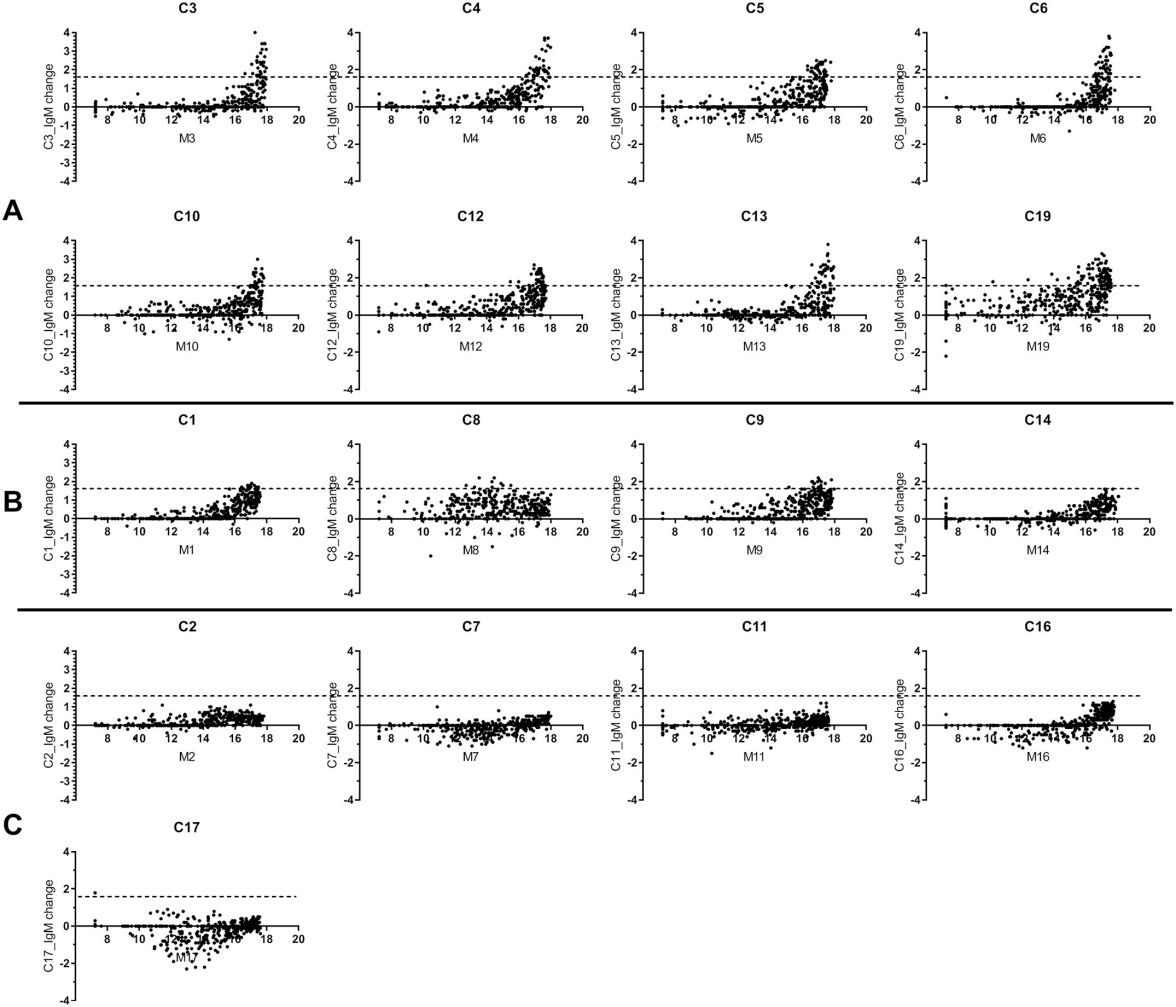
Effects of adding maternal serum to cord serum samples. Cord sera were profiled with and without adding 0.1% matched maternal serum; IgM antibodies were profiled on glycan microarray. X-axis: IgM signals in maternal (in Log2 scale); Y-axis: The change of antibody signals in maternal-spiked cord relative to cord alone (in Log2 scale). Dotted line indicates a 3-fold cutoff. Panel A: Cord samples with high purity (i.e. many points above the threshold); panel B: cord samples estimated to have 0.03–0.1% maternal contamination level (i.e. a few points above the threshold); panel C: cord samples estimated to have >0.1% maternal contamination level.

We next performed the spike-in experiment for our 17 cord/maternal serum pairs. Pairs 18 and 20 were not evaluated in this experiment due to high IgA levels in the cord sample. Pair 15 was also excluded since the maternal sample had IgA levels of only 1.5 μg/mL. We speculated that this sample was likely mis-labeled since the IgA level was 1000-fold lower than expected. After profiling all 17 cord/maternal serum pairs, 8 of the samples produced many 3-fold or greater changes and at least ten 4-fold or greater changes upon adding 0.1% matched maternal serum (Fig 4A). Thus, we estimated that these samples have less than ~0.033% maternal serum contamination and were essentially pure. Five samples (Fig 4B) were deemed to be contaminated by ~0.033–0.1% maternal serum. These samples were considered borderline; the vast majority of signals were likely due to the fetus, but some maternal signals could be present. Four samples were estimated to have > 0.1% maternal contamination (Fig 4C). Any sample with greater than 0.033% maternal contamination was not used for further analysis.

### Evaluation of cord blood anti-glycan IgM repertoires

**Fig 5:**
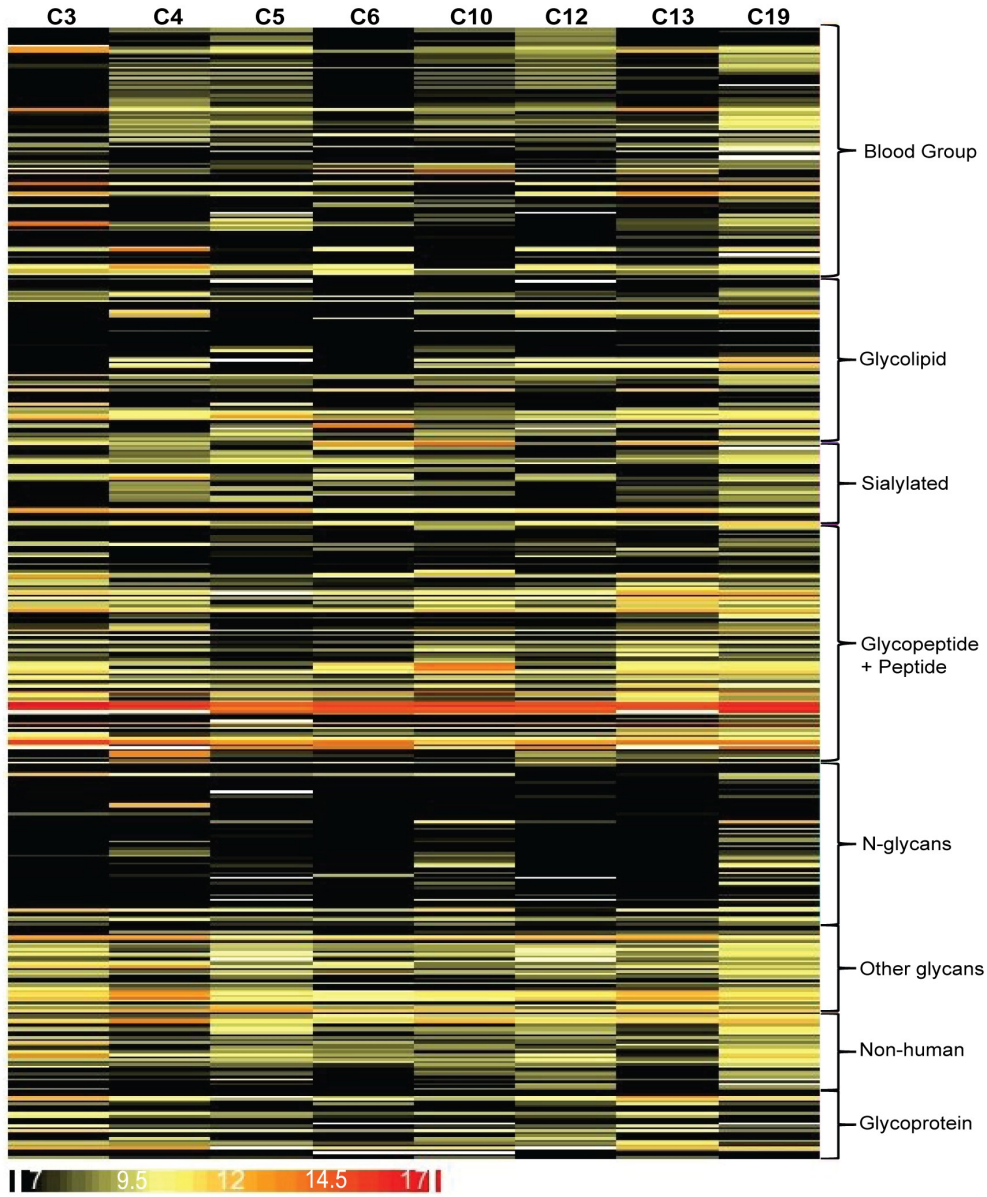
Heatmap of cord IgM signals. Each row represents a cord sample and each column represents an antigen on the microarray. Antigens are grouped by family. The magnitude of IgM signals is denoted by different colors: black (no/low signal), yellow (signals with medium intensity) and red (signal with high intensity). A positive signal is defined if the fluorescent intensity is 2-fold above background (≥ 8.8 at Log2 scale).

For the 8 samples with an estimated purity of over 99.97%, cord sera contained a variety of anti-glycan IgM antibodies that recognized a broad spectrum of carbohydrate antigens (62% of 480 array components; controls were excluded) on our glycan microarray. The heatmap in Fig 5 provides an overview of cord IgM profiles. Individual samples each showed 128–227 unique anti-glycan IgM signals. Looking at individual antibodies, we detected IgM antibodies targeting 152 array components (30%) in at least half of the cord samples, and among them, 46 IgM signals were found in all 8 samples (see Table A in S1 File). The 8 cord samples had an average of 73 array signals over 1000 RFU (range = 53–113 signals over 1000; not including controls). As mentioned previously, we would not expect any signals over 1000 from maternal contamination of 0.033% or less. The IgM profiles for the 8 pure cord samples were evaluated on two different version of our microarray, one with 503 components and another with 411 components. The resulting IgM profiles were highly consistent (Pearson correlation coefficient = 0.89–0.96; see also Figure B in S1 File), indicating reproducible results from separate experiments carried out approximately 8 months apart on different arrays.

Table 1 lists the most frequently observed cord IgM antibodies. One of the highest and most consistent signals observed on our array was to one of the non-glycan components, dinitrophenylated albumin (DNP-BSA). Anti-DNP IgM are abundant in healthy adults and have been previously reported to be natural antibodies.[35, 36] The detection of anti-DNP IgM indicates that our microarray and assay are capable of detecting natural antibodies that are present in cord sera.

One common trend was the presence of antibodies that bound tumor associated antigens. For example, all 8 cord samples had IgM to the Tn antigen, a tumor associated carbohydrate antigen wherein a terminal GalNAc residue is linked to Ser/Thr of a protein. The Tn antigen can occur within a variety of peptide sequences, and it can be found in clusters wherein 2 or more Ser/Thr residues contain a GalNAc. Our array contains 55 Tn-containing glycopeptides, including different sequences and clusters, and 44 of them were recognized by at least one cord IgM. The antibodies bound best to a 19 amino acid MUC1 tandem repeat sequence containing a GalNAc on the threonine within the PDTRP region of the repeat (MUC1-Tn8: GVTSAPDT(GalNAc-α)RPAPGSTAPPA), followed by a MUC1 glycopeptide wherein the GalNAc was positioned on the threonine within the GSTAP region of the repeat (MUC1-Tn15: GVTSAPDTRPAPGST(GalNAc-α)APP). Antibodies were also observed to the non-glycosylated MUC1 tandem repeat; however, the signals were 2–30 fold lower than the two glycopeptides, highlighting the importance of glycosylation. The anti-MUC1-Tn8 IgM signals in two cord samples were at least 5-fold higher than their corresponding maternal samples (see Figure D in S1 File). Some IgM signals are higher in cord than maternal samples, which further indicates that they are produced in the fetus rather than arising from maternal contamination. Antibodies were also observed to a variety of the other Tn glycopeptides. Two cord samples had fairly high antibody signals to clusters of GalNAc-Ser [Ac-Tn(Ser)-Tn(Ser)-Tn(Ser)-G]; interestingly, the signals were approximately 30-fold lower to analogous clusters of GalNAc-Thr [Ac-Tn(Thr)-Tn(Thr)-Tn(Thr)-G] indicating that the amino acid core plays a significant role in recognition.

Antibodies to various other tumor-associated antigens were also observed in the cord samples. All 8 cord samples had antibodies to alpha-fetoprotein.[37] For 2 cord samples, the IgM signals were larger than the corresponding maternal samples. Five of the 8 cord samples had antibodies to a MUC4 glycopeptide containing a cluster of two TF antigens, another well-known tumor associated carbohydrate antigen.[38] Half of the cord samples had antibodies to the Lewis C antigen. IgM were also observed to ganglioside GD2, stage-specific embryonic antigen 4 (SSEA4), and stage specific embryonic antigen 5 (SSEA5, also known as Gb5).

Beyond tumor associated antigens, cord IgM antibodies recognized various non-human antigens. All 8 cord samples had antibodies to chitotriose, a trisaccharide found on a variety of pathogens.[39] Antibodies were also observed to other microbial glycans, including rhamnose, Glcβ1-4Glc, and the Forssman antigen. IgM were observed to one plant glycan, a pentasaccharide composed of five arabinose monosaccharides with α1–5 linkages. Although adults often have very high antibody levels to the α-Gal antigen, the cord samples had very low or no antibodies to this foreign carbohydrate. The lack of IgM to α-Gal in cord blood is consistent with a previous report.[40]

The cord IgM repertoire also recognized a broad range of mammalian glycans, including blood group antigens, glycolipids and glycoproteins. All 8 cord samples had antibodies to the glycolipid P1 antigen. We found high signals in 1–2 cord samples to a variety of glycans, including dimeric LacNAc, monofucosyl lacto-*N*-hexaose I, core 3, Lac-di-NAc (LDN), and GalNAcα1-3Gal. Several cord samples had strong IgM signals to the blood group A trisaccharide or the blood group B trisaccharide when attached to a flexible linker. Measurable signals were also observed to blood group A and B when attached to a carrier chain, as occurs in humans, but they were found in fewer samples and at lower levels. Four cord samples had IgM to blood group H4 and H5.

### Systems level analysis of cord blood and maternal anti-glycan IgM antibody profiles

**Fig 6:**
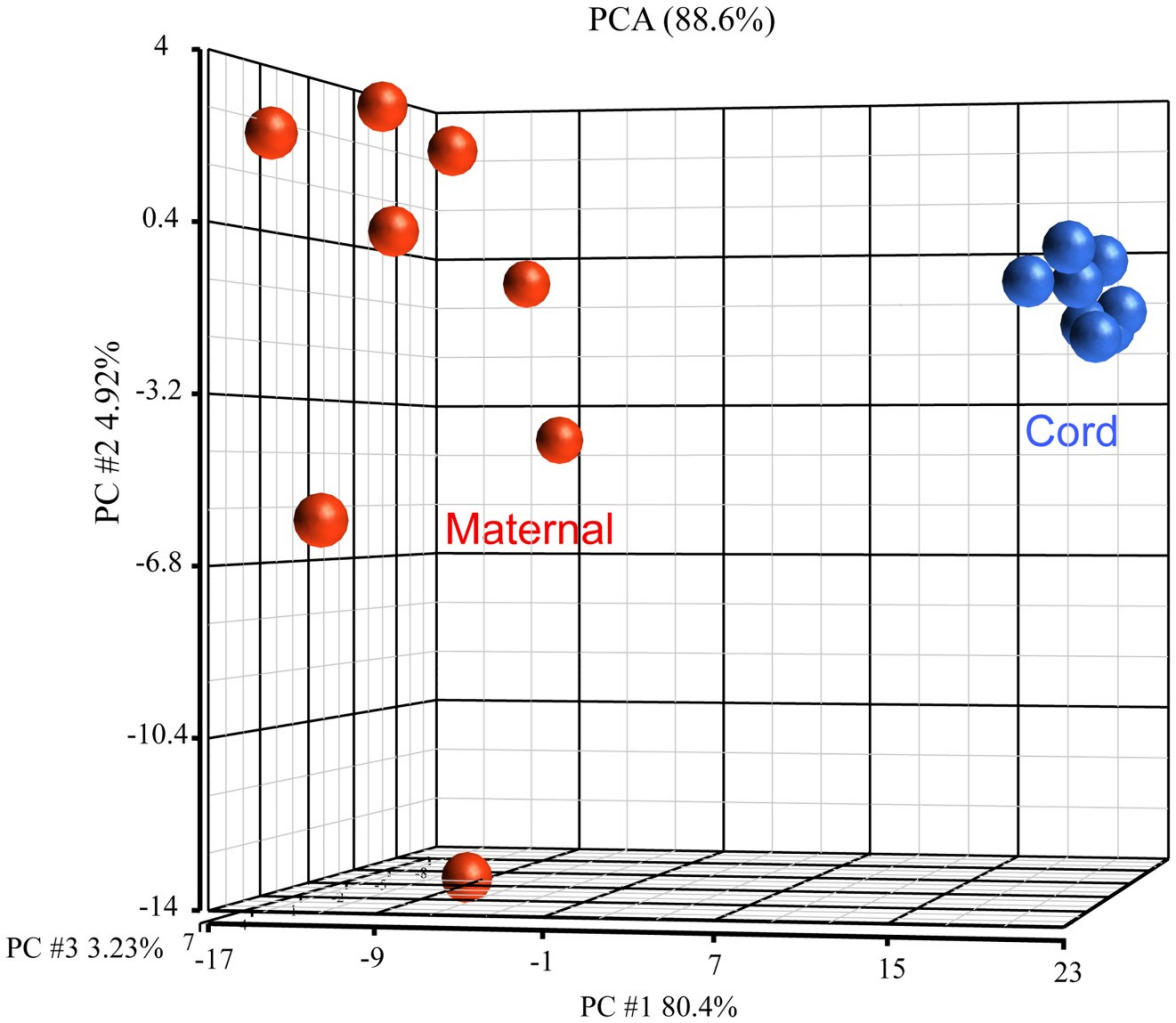
Principle component analysis of cord IgM *vs*. maternal IgM. PCA analysis of cord and maternal RFU signals on the array. Blue are cord samples. Red are maternal samples.

We next evaluated relationships between cord IgM and maternal IgM repertoires using a combination of hierarchical clustering, principal component analysis, and correlation matrices. We wanted to determine if cord IgM repertoires were more similar to other cord blood samples or to matched maternal sera. IgM profiles from cord serum were not well correlated with maternal serum (Pearson correlation coefficient range = 0.01–0.42; see also Figure C in S1 File). Instead, principal component analysis showed the cord and maternal IgM were clearly separated (Fig 6A). The Pearson coefficient within cord and maternal samples were 0.66 ± 0.07 and 0.73 ± 0.06, respectively. Moreover, the group of cord samples produced a very tight cluster while the maternal samples were more distributed. Hierarchical clustering also separated cord and maternal serum samples into two distinct groups.

**Fig 7:**
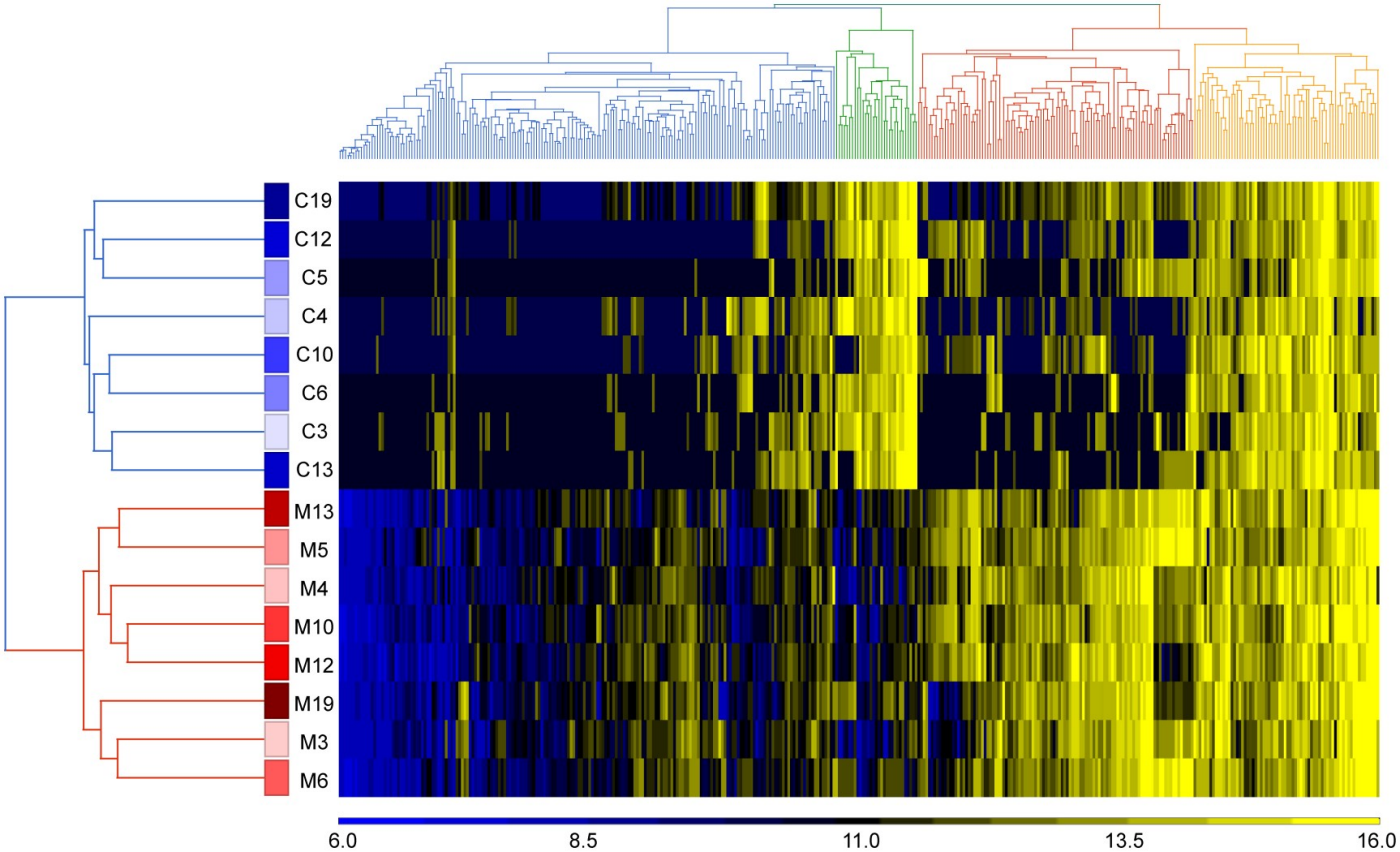
Clustering of quantile normalized cord and maternal IgM. Hierarchical clustering was carried out for both samples and array components using average linkage and Euclidean distance metric. Cord and maternal IgM separated into two distinct groups. Data for each serum sample is presented in rows. Samples are labeled with “C” for cord or “M” for maternal followed by the sample number. Data for each glycan is shown in columns.

The signal intensities of cord IgM were significantly lower than adults, with the mean signal intensity of 153±82 RFU vs. 544±215 RFU for the maternal IgM. So, we also compared cord and maternal profiles after quantile normalizing all signals to account for differences in signal strength. Even with quantile normalization, the cord samples still clustered together and further away from all the maternal samples (Figs 6B and 7).

## Discussion

Human serum contains a rich and diverse collection of anti-glycan antibodies. Understanding the origins and development of these antibodies can reveal insights into immune system function, origins of autoimmune reactions, and induction of productive immune responses during vaccination. Many anti-glycan antibodies share features consistent with natural antibodies; however, it is unclear which, if any, are natural antibodies. So, our goal in this study was to investigate whether anti-glycan antibodies present in human serum are natural antibodies.

Our approach employed glycan antigen microarrays to profile the repertoire of IgM antibodies present in serum at birth. Since maternal IgM cannot cross the placenta, IgM antibodies present in cord serum are considered natural antibodies. While there have been a few studies examining anti-glycan antibodies at birth, they have investigated only a small number of glycan antigens and there is little consensus whether anti-glycan antibodies exist at birth.[5] We exploited glycan microarray technology to address this fundamental question. Glycan microarrays have been used previously to profile serum anti-glycan antibodies in adult serum (for some recent examples, see [10, 11, 23, 34, 41–51]; for a recent review, see [52]), but glycan microarrays have not been used to study the population(s) of serum anti-glycan antibodies in cord blood.

When assaying cord blood, contamination with maternal blood complicates the analysis.[29, 30] While this problem is well known, detecting maternal contamination is challenging, especially in serum which contains no cells and little or no DNA. For serum samples, the primary method of detecting contamination involves measuring IgA levels in cord blood.[30] Newborns produce very low levels of IgA, whereas the mean concentration of IgA in maternal blood is 2–3 mg/mL. Therefore, unusually high IgA levels in cord blood indicate maternal contamination. While this method remains the standard in the field, there are some caveats. First, the amount of IgA produced by fetuses prior to birth is not well-defined. For example, reported mean IgA values measured in infants have ranged from 5–55 μg/mL.[30, 53] Since the concentration of IgA varies significantly among infants, IgA levels produced by a single infant are difficult to assess. Second, the acceptable amount of contamination depends on the analyte(s) of interest. For these reasons, the optimal cutoff for defining maternal contamination varies considerably from study to study and is still a matter of debate. Depending on the study, the cutoff has varied from 10–100 μg/mL.[30]

To detect potential contamination and evaluate potential IgA cutoffs, we measured IgA levels in the cord samples used in this study. In addition, we profiled each of the cord samples, along with matched maternal serum, on our glycan microarray. Three cord samples had IgA levels between 10–25 μg/mL, and these had unusually high correlations with the matched maternal serum, i.e. Pearson correlation coefficients of ~0.8 between cord and maternal IgM. Several other cord samples also had high correlations with their corresponding maternal serum, even though the IgA levels were below 10 μg/mL. We hypothesized that the maternal IgM present in these cord samples were contributing significantly to the signals being measured on our array, leading to a high correlation.

To evaluate this further, we spiked maternal serum into cord samples and evaluated the effects on our microarray. We found that maternal contamination as low as 0.1% could significantly change cord IgM profiles to carbohydrate antigens. Therefore, we needed a much more sensitive assay to detect maternal contamination than measuring cord IgA levels.

To address this challenge, we developed a microarray-based approach to detect maternal contamination. The method involves profiling cord samples on their own and then reassaying them after adding 0.1% maternal serum. The two array profiles are then compared to determine if adding maternal serum significantly alters that array profiles. This method is highly sensitive and can detect contamination levels as low as 0.03%. Using this method, we found that a substantial portion of the cord samples (58%) had unacceptable levels of maternal contamination and that only 8 of the cord samples (42%) had an estimated purity greater than 99.97% (essentially pure, at least within the limits of our assay). Our results demonstrate that unacceptable levels of maternal contamination are common when profiling anti-glycan IgM.

In the second phase of the study, we analyzed carbohydrate-binding IgM antibodies present at birth for the 8 cord samples with high purity. These cord samples contained abundant glycan-specific IgM antibodies recognizing a broad spectrum of carbohydrate antigens (see Table 1 and Table A in S1 File). The observed antibodies could be grouped into two families: antibodies that recognize human glycans and antibodies that recognize foreign glycans.

A variety of IgM recognized non-human glycans that are present on bacteria, fungi, and other microbes. For example, all 8 cord samples had high levels of IgM to chitotriose, a trisaccharide epitope found in chitin. Chitin is a polysaccharide component of the cell walls of many fungi and other microbes, and anti-chitin IgM may play a role in host defense. Cord serum has previously been found to possess IgM to β-glucan and chitosan (chitin that is about 80% deacetylated yielding a polymer this is about 80% glucosamine and 20% GlcNAc).[15] These antibodies were shown to protect mice from fungal infection.[15] Most cord samples also had high levels of IgM to rhamnose, a carbohydrate found in bacterial polysaccharides. Antibodies to non-human glycans may be part of our natural defense against invading pathogens and/or they might influence the composition of the human microbiome.

In addition to non-human glycans, we also observed IgM to mammalian glycans. Some of these antibodies recognized various glycans found in normal adult tissue, such as LacNAc, Lac-di-NAc (LDN), 6’Neu5Ac-LacNAc, GT2, lacto-N-tetraose (LNT), GlcA-LNT, blood group H4, blood group H5, and the core 3 *O*-glycan. Other antibodies recognized what are referred to as oncofetal antigens or tumor-associated carbohydrate antigens (glycans that have high expression during development and in various cancer cells but have low expression in healthy adult tissue).[38, 54] For example, we found abundant IgM antibodies to glycopeptides containing the tumor-associated Tn antigen (GalNAc alpha linked to a serine or threonine). The highest signals were to Tn displayed on a MUC1 tandem repeat sequence. Cord IgM also bound several other tumor-associated carbohydrate antigens such as the ganglioside GD2, stage-specific embryonic antigen 4 (SSEA4), and stage specific embryonic antigen 5 (SSEA5, also known as Gb5).

Natural antibodies to self-antigens have been observed previously.[1–4] These antibodies recognize human antigens as part of the normal process of removing apoptotic cells and cellular debris. They are thought to help control inflammation and autoimmunity.[4] While these autoantibodies are considered beneficial, they may give rise to disease-causing autoantibodies either through overexpression or via acquisition of higher affinity, altered selectivity, and/or altered effector functionality.

The presence of antibodies to tumor-associated carbohydrate antigens suggests that some natural antibodies may serve as part of our natural immuno-surveillance against cancer, too. Previously, Vollmers [55, 56] has identified IgM in adults that bind tumors in a glycan dependent manner; however, the specific glycan epitopes were not determined. These antibodies had relatively few mutations from germline sequences and were postulated to be natural antibodies. In addition, natural antibodies to tumor-associated protein antigens have also been observed.[57] In a recent study, B1-cell produced natural IgM antibodies provided protective effects in a mouse model of metastatic cancer involving spread to the peritoneal cavity.[58]

Antibodies to blood group A (BG-A) and B (BG-B) antigens are of critical importance in blood transfusions and organ transplants. Antibodies to these glycans are thought to develop over the first few months of life, and once they are present they can induce complications for mismatched transfusions and transplants. So, elucidating the origins and development of these antibodies is of vital interest for therapeutic strategies. The BG-A and BG-B determinants are defined as trisaccharides [BG-A = GalNAcα1-3(Fucα1–2)Gal; BG-B = Galα1-3(Fucα1–2)Gal]. In nature, however, these trisaccharides are attached to one of six carrier glycan chains, producing six tetrasaccharides for each (BG-A1 through A6, and BG-B1 through B6). We detected substantial cord IgM to the blood group A trisaccharide and the BG-B trisaccharide when attached to a linear, flexible linker. In most cases, there was little or no binding to the BG-A or BG-B trisaccharide when presented on a carrier chain. In a few cord samples, we observed low but measurable IgM to a small subset of BG-A and BG-B tetrasaccharides; however, the levels were 50–100 times lower than those found in adults. Discrimination based on the carrier chain has been observed previously for serum antibodies and monoclonal antibodies to BG-A and BG-B.[48, 59] Recognition of small glycan fragments but not the full, natural glycans has been observed previously.[60]

At present, we do not know why the immune system produces antibodies that bind BG-A or BG-B trisaccharides alone but not when attached to a carrier glycan chain. In our previous work on monoclonal antibodies to BG-A, we found that the carrier chain did not provide any additional affinity beyond recognition of the trisaccharide. Instead, the carrier chain acted like a selectivity filter- either allowing binding to BG-A or blocking/inhibiting it.[59] Taken together, these data suggest a model wherein the immune system encodes natural antibodies that can bind the trisaccharide tightly but have one or more amino acid residues that inhibit/block binding when the trisaccharide is attached to a carrier glycan. In this model, the immune system would be primed to produce antibodies to natural BG-A and BG-B antigens, once the blocking residues are mutated to allow recognition of BG-A/BG-B on natural carrier chains. Alternatively, BG-A and BG-B trisaccharide-binding antibodies may recognize BG-A and BG-B determinants on a carrier that has not yet been discovered (the array contains all 12 known BG-A and BG-B tetrasaccharides).

Although natural antibodies are thought to have broad specificity,[6] the profiles we observed suggest that some cord IgM antibodies have high selectivity. Cord samples C3, C5, and C13 had abundant IgM to the pentasaccharide Neu5Acα2-6Galβ1-4GlcNAcβ1-3Galβ1-4GlcNAc [6’Neu5Ac-LacNAc (dimeric);]; however, there were little or no signals to several closely related glycans including Neu5Acα2-6Galβ1-4GlcNAc (6’Neu5Ac-LacNAc), Neu5Acα2-6Galβ1-3GlcNAc (6’Neu5Ac-LeC), Neu5Acα2-6GalNAcβ1-4GlcNAc (6’Neu5Ac-LDN), and Neu5Acα2-3Galβ1-4GlcNAcβ1-3Galβ1-4GlcNAc [3’Neu5Ac-LacNAc (dimeric)]. As another example, cord sample C19 had very high signals to the glycopeptide P-D-T(GalNAcα)-R-P (44,453 RFU). Signals to the non-glycosylated peptide P-D-T-R-P (1600 RFU) and the shorter glycopeptide D-T(GalNAcα)-R (300 RFU) were significantly lower. Thus, we conclude that IgM recognizing P-D-T(GalNAcα)-R-P can distinguish closely related structures.

We also conducted a systems level comparison of maternal and cord serum IgM repertoires using hierarchical clustering and principal component analysis. Prior studies on antibodies to protein antigens found that cord IgM repertoires are more similar to each other than to maternal IgM repertoires.[12, 13] In addition, they found that cord IgM were tightly clustered whereas maternal profiles were more unique to each individual. At the outset, we did not know if anti-glycan IgM would follow a similar pattern or have distinct properties from anti-protein IgM. In this study, we found that anti-glycan IgM repertoires at birth have these same characteristics, which suggests that the development and regulation of natural antibodies to proteins and glycans are coordinated rather than being dependent on the class of antigen being recognized. However, the mechanisms that give rise to the repertoire of natural antibodies are not well understood. As a reviewer noted, the production of similar IgM repertoires from genetically unrelated fetuses is unlikely to arise via random recombination of antibody genes. A more likely scenario for shared IgM repertoires, therefore, is that natural antibodies are selected during B cell development through a common mechanism in human fetuses. Additional studies will be need to more fully assess this possibility.

Several limitations of our study should be noted. Our microarray only contains a subset of naturally occurring glycans. So, care should be taken when making conclusions regarding anti-glycan antibodies not detected on our array. The microarray assay used to detect maternal contamination also requires access to matched cord and maternal serum as well as additional work (i.e. evaluation of cord with and without addition of maternal serum). Because of the high proportion of cord samples that were contaminated with maternal serum, our analysis of cord IgM repertoires included a relatively small number of samples. While analysis of additional samples will be helpful to further evaluate our findings, evaluation of 8 samples on a glycan microarray with 500 array components provides a large dataset to assess anti-glycan IgM repertoires at birth. In addition, it is of similar scope as prior studies using protein microarrays.[12, 13] Nevertheless, additional studies will be helpful for further validating our findings.

In summary, high-throughput analysis of serum IgM using a glycan microarray has enabled several key findings. We demonstrated that contamination of cord blood with maternal blood is especially problematic when studying anti-glycan antibody repertoires. In addition, we disclose a highly sensitive assay for detecting maternal contamination. Finally, we demonstrate that humans have a diverse assortment of anti-glycan IgM at birth, providing further evidence that many anti-glycan antibodies are likely natural antibodies. Overall, the results provide new insights into the origins of anti-glycan antibody repertoires and the development of the immune system.

## Supporting information

S1 File Supporting figures and tables. (PDF)

S2 File Full array data. (XLSX)
