# Supplementary material for: Anti-glycan IgM repertoires in newborn human cord blood
Source: PLoS One. 2019 Jul 31;14(7):e0218575. doi: 10.1371/journal.pone.0218575 (PMC6668783; doi:10.1371/journal.pone.0218575)
Supplement: S1 File — (PDF) [file pone.0218575.s001.pdf]

S1 File

Supporting Information

For

Anti-Glycan IgM Repertoires in Newborn Human Cord Blood

Li Xia[1], Jeffrey C. Gildersleeve[1],

[1]Chemical Biology Laboratory, Center for Cancer Research, National Cancer Institute, National Institutes of Health, Frederick, MD

Table of contents

Table A. List of cord IgM detected in at least 50% of cord samples

Figure A. Representative images of printed glycan microarrays and cord IgM profiling results.

Figure B. Representative scatter plots of IgM profiles within cord, within maternal, and between cord and maternal samples.

Figure C. Comparison of cord IgM profiles on A411 vs. A503.

Figure D. Anti-MUC1-Tn8 IgM signals are higher in cord than maternal sera.

Table A. List of cord IgM detected in at least 50% of cord samples

Category	Abbreviation[1]	Mean[2]	Max[2]	# of samples with IgM ≥8.8 (3-fold of bkg)	# of samples with IgM ≥10 (7-fold of bkg)
Blood Group	BG-B (Dextra) - 13	10.9	13.7	8	5
Blood Group	BG-H2 - 16	10.1	11.3	5	4
Blood Group	BG-H4- 15	10.3	13.4	6	4
Blood Group	BG-H5-19	10.4	13.5	6	4
Blood Group	MFLNH I - 11	10.8	14.2	7	6
Glycolipid	Gala1-4Galb - 11	10.7	11.8	8	7
Glycolipid	Gb5/SSEA3 - 12	10.1	11.8	5	4
Glycolipid	**GD2-Sp - 04**	9.6	12.5	5	4
Glycolipid	**GD2-Sp - 10**	9.9	13.1	5	4
Glycolipid	GQ2-Sp - 06	9.8	12.2	5	5
Glycolipid	GT2-Sp - 03	9.2	11.7	5	4
Glycolipid	GT2-Sp - 08	9.7	12.4	5	4
Glycolipid	Lac - 33	10.0	10.9	8	5
Glycolipid	Lac-C5 - 14	10.6	12.8	8	5
Glycolipid	P1 - 09	11.6	14.2	8	7
Sialylated	3'Neu5Ac(9Ac)-LeC-Sp - 12	10.0	11.2	7	5
Sialylated	6'Neu5Ac-LacNAc (dimeric)-Sp - 05	11.3	12.6	8	8
Sialylated	6'Neu5Ac-LacNAc (dimeric)-Sp - 13	12.1	13.7	8	8
Sialylated	CT/Sda-Sp - 05	10.1	11.9	8	4
Sialylated	CT/Sda-Sp - 13	10.5	12.0	8	5
Glycopeptide+peptide	**Muc1-Tn15**	14.9	16.1	8	8
Glycopeptide+peptide	**Muc1-Tn8**	15.3	16.2	5	5
Glycopeptide+peptide	**Muc1**	13.5	14.6	5	5
Glycopeptide+peptide	GTSSA-TF(Ser)-TF(Thr)-GHATPLPVTD	10.6	13.0	8	4
Glycopeptide+peptide	Ac-APGS-Tn(Thr)-APPA-G-03	11.5	12.9	7	7
Glycopeptide+peptide	Ac-A-Tn(Thr)-S-G - 23	10.7	12.3	8	6
Glycopeptide+peptide	Ac-G-S-T(Tna)A-P-G-Hex-19	10.6	11.8	8	6
Glycopeptide+peptide	Ac-P-Tn(Thr)-T-G - 08	10.3	11.6	8	5
Glycopeptide+peptide	Ac-P-Tn(Thr)-T-G - 22	11.9	13.0	8	8
Glycopeptide+peptide	Ac-S-Tn(Thr)-A-G - 22	11.2	12.4	8	7
Glycopeptide+peptide	Ac-S-Tn(Thr)-G-G - 19	9.9	11.3	6	5
Glycopeptide+peptide	Ac-Tn(Ser)Tn(Ser)Tn(Ser)-G - 03	9.5	13.6	5	5
Glycopeptide+peptide	Ac-Tn(Ser)-Tn(Ser)-Tn(Ser)-G - 16	10.7	14.3	6	5
Glycopeptide+peptide	Ac-Tn(Ser)Tn(Ser)Tn(Ser)-G - 27	10.8	14.3	8	5
Glycopeptide+peptide	Ac-Tn(Thr)-G - 21	11.0	12.0	8	8
Glycopeptide+peptide	Ac-V-Tn(Thr)-S-G - 19	10.2	11.8	8	5
Glycopeptide+peptide	AzHex-PD-Tn(Thr)-RP-NH2-07	14.0	15.4	8	8

Category	Abbreviation[1]	Mean[2]	Max[2]	# of samples with IgM ≥8.8 (3-fold of bkg)	# of samples with IgM ≥10 (7-fold of bkg)
Glycopeptide+peptide	AzHex-SAPD-Tn(Thr)-RPAP-NH2-07	15.5	16.8	8	8
Glycopeptide+peptide	AzHex-VTSAPD-Tn(Thr)-RPAPGS-NH2-06	16.1	17.3	8	8
Glycopeptide+peptide	Ac-APGSTAPPA-G-05	10.9	12.4	6	4
Glycopeptide+peptide	Ac-APGSTAPPA-G-14	13.0	14.4	8	8
Glycopeptide+peptide	Ac-GSTAP-G-15	10.8	11.7	8	7
Glycopeptide+peptide	AzHex-P-D-T-R-P-NH2-07	10.7	11.6	8	7
Glycopeptide+peptide	AzHex-S-A-P-D-T-R-P-A-P-NH2-07	13.4	15.5	8	8
Glycopeptide+peptide	AzHex-V-T-S-A-P-D-T-R-P-A-P-G-S-NH2-07	13.9	15.4	8	8
Glycopeptide+peptide	Ac-S-Thr(core 3)-S-G - 21	10.7	13.8	8	5
Other Glycans	GalNAc-a - 22	10.1	11.1	7	4
Other Glycans	LDN-Sp - 14	10.5	13.0	7	4
Other Glycans	Glc-b - 23	9.9	11.6	7	4
Other Glycans	GlcA-LNT-05	11.4	12.6	8	8
Other Glycans	GlcA-LNT-16	13.3	14.1	8	8
Other Glycans	3'GN-LacNAc (dimeric)-Sp - 06	12.1	14.0	8	8
Other Glycans	3'GN-LacNAc (dimeric)-Sp - 14	11.3	13.1	8	8
Other Glycans	GlcNAc-b -21	10.2	11.0	8	5
Other Glycans	GNLacNAc-Sp - 06	10.7	12.0	8	7
Other Glycans	GNLacNAc-Sp - 16	12.0	14.2	8	8
Other Glycans	LNT-2-Sp - 15	12.1	13.1	8	8
Other Glycans	LNT-Sp - 15	9.9	14.0	5	4
Other Glycans	LacNAc (dimeric)-Sp - 16	10.3	13.2	7	5
Other Glycans	LacNAc (trimeric) - 08	11.0	12.7	8	7
Other Glycans	3'-sulpho-LeX - 15	10.3	12.1	7	4
N-glycan	Ma6Ma6-05	10.3	11.7	7	6
Foreign antigens	Cellotriose - 13	9.9	12.0	6	4
Foreign antigens	Chitotriose - 08	11.6	13.5	8	7
Foreign antigens	Chitotriose - 20	12.0	13.9	8	8
Foreign antigens	Rha-a - 18	10.3	13.7	7	5
Foreign antigens	iGb5-15	10.9	14.5	7	4
Glycoprotein	**Alpha-fetoprotein**	11.1	14.0	7	5
Glycoprotein	BSM	10.8	12.2	8	6
Glycoprotein	fetuin (human)	12.1	12.9	6	6
Glycoprotein	Ovalbumin (ox)	10.1	11.4	7	5

[1]components in bold are known tumor-associated antigens. [2]All signals are on a $\log_2$ scale. Full descriptions for each array component abbreviation can be found in the Supporting Excel File.

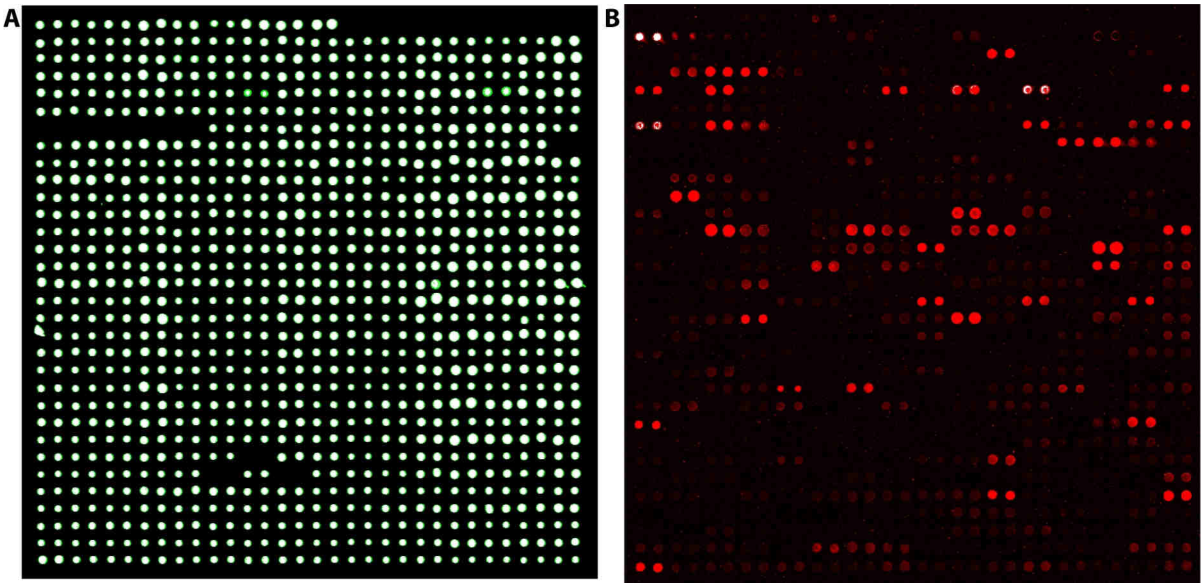

Figure A. Representative images of printed glycan microarrays and cord IgM profiling results. (A) Pre-assay: Representative image of a printed microarray before running an assay is shown. In this array, a total of 503 array components were printed in duplicate in a 32 rows × 32 columns format with a pitch of 140 μm. Atto 532 dye (Sigma) at 0.7 μg/mL was added to the print buffer to help visualize the printed spots. The printed slides were imaged in GenePix 4000B microarray scanner (Molecular Devices) or InnoScan 1100AL fluorescence scanner (Innopsys). In this example, 9 missing components were identified from the array. They were excluded from further data analysis. Atto532 signals are green or white if saturated. (B) Post-assay image: A representative image of IgM profiling in cord samples is shown. The cord samples were profiled at 1:5 dilution and cord IgM bound to the microarray were detected with DyLight 649 anti-human IgM (red).

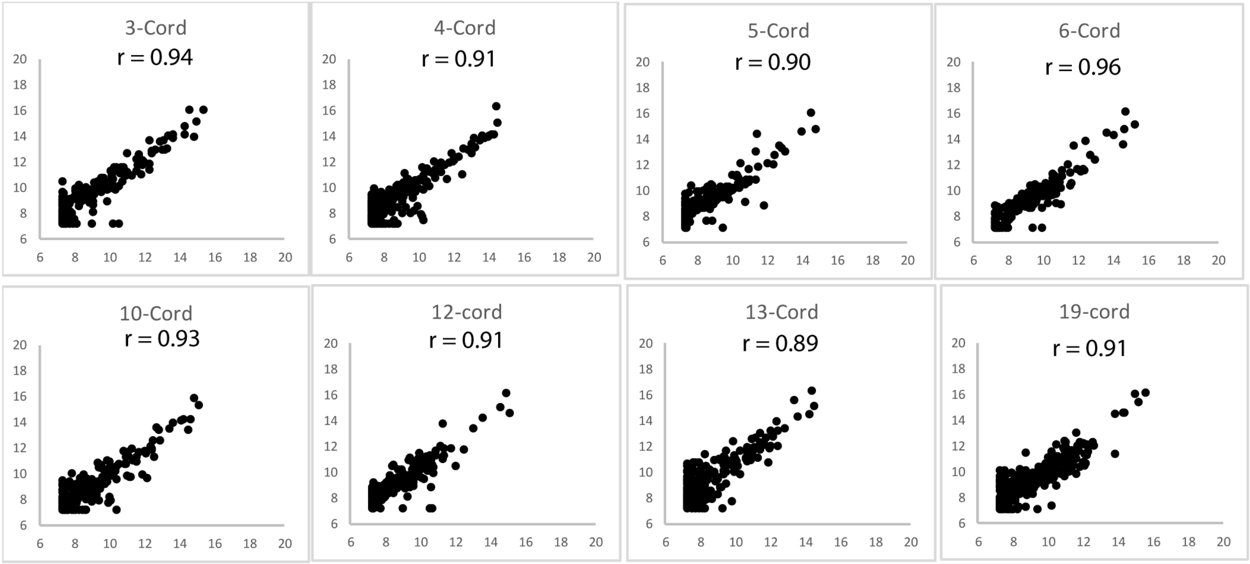

Figure B. Comparison of cord IgM profiles on A411 vs. A503. IgM profiles on A411 (x-axis) vs. A503 (Y-axis) Fluorescence intensity of IgM signals were Log-transformed (base 2). *r*: Pearson coefficient.

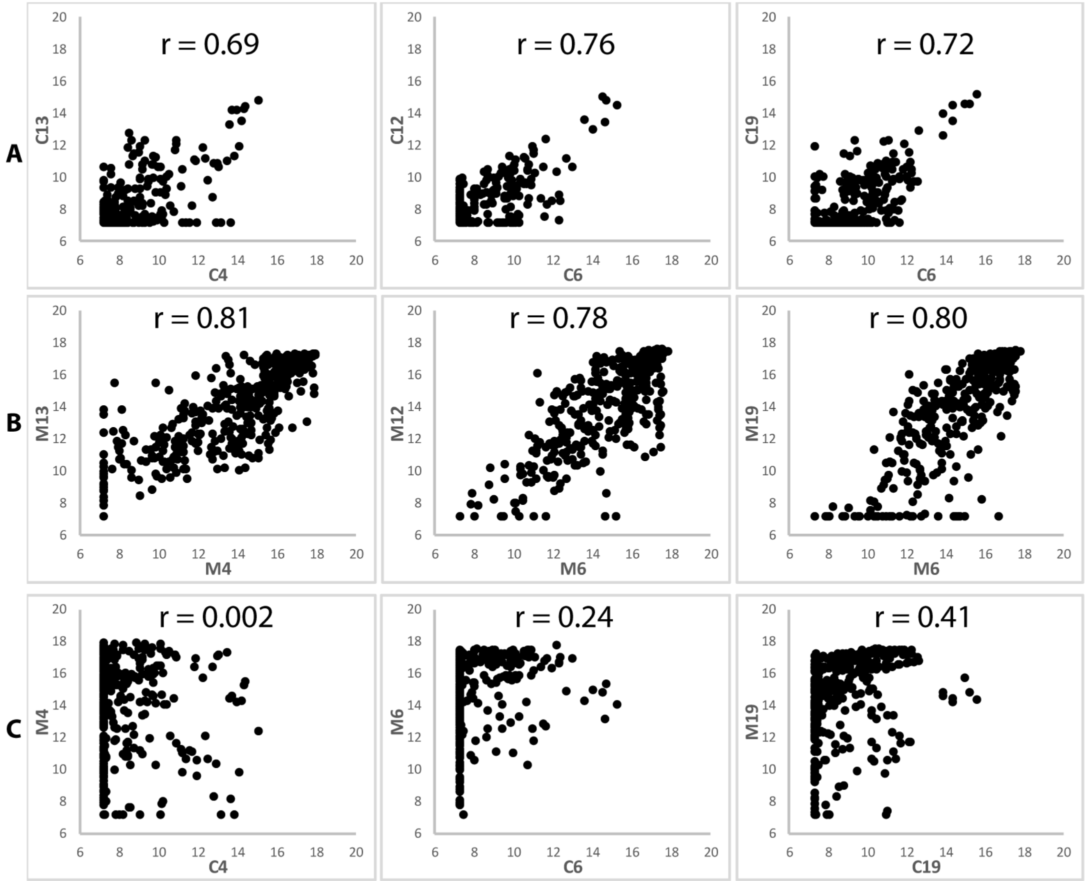

Figure C. **Representative scatter plots of IgM profiles within cord, within maternal, and between cord and maternal samples.** IgM profiles within cord samples (A) and within maternal samples (B) are similar with highly correlated Pearson coefficients. In comparison, IgM profiles between paired cord-maternal samples (C) are dissimilar with low correlations. Fluorescence intensity of IgM signals were Log-transformed (base 2). *r*: Pearson coefficient.

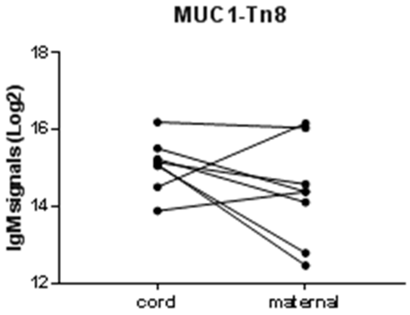

Figure D. Anti-MUC1-Tn8 IgM signals are higher in cord than maternal sera. Fluorescence intensity of IgM signals were Log-transformed (base 2).
